# Electrospray Polytetrafluoroethylene Membranes: Effect of Composition and Heat Treatment on Mechanical Properties and Chemical Resistance

**DOI:** 10.3390/polym18202504

**Published:** 2026-10-13

**Authors:** Evgeniy Melnik, Vladimir Botvin, Kirill Shirin, Valery Svetlichnyi, Aleksandra Golubovskaya, Vyacheslav Buznik, Andrey Yaroslavtsev, Evgeny Bolbasov

**Affiliations:** 1 Additive Technologies Center, National Research Tomsk Polytechnic University, Tomsk 634050, Russia; eymelnik@tpu.ru; 2 Faculty of Chemistry, National Research Tomsk State University, Tomsk 634050, Russia; v.v.botvin@gmail.com (V.B.); shirinkn86@gmail.com (K.S.); buznikv@list.ru (V.B.); 3 Laboratory of Advanced Materials and Technology, National Research Tomsk State University, Tomsk 634050, Russia; v_svetlichnyi@bk.ru (V.S.); aleksandra.golubovskaya@mail.ru (A.G.); 4 Kurnakov Institute of General and Inorganic Chemistry of the Russian Academy of Sciences, Moscow 119991, Russia; yaroslav@igic.ras.ru

**Keywords:** polytetrafluoroethylene membranes, electrospray, heat treatment, mechanical properties, chemical resistance

## Abstract

Electrospray is a promising method for fabricating semipermeable polytetrafluoroethylene (PTFE) membranes. PTFE membranes are attractive for filtration and separation in harsh environments, but their mechanical properties depend strongly on crystallinity and thermal history. In this work, PTFE/poly(ethylene oxide) (PEO) membranes were fabricated by electrospraying aqueous solutions with PTFE/PEO ratios of 98.8/1.2, 97.5/2.5, 95/5, and 90/10 wt.%. Increasing the PEO content increased the solution viscosity from 43–78 to 124 mPa·s and caused a transition from sphere-based to mixed fiber–sphere morphology. After sintering at 330 °C, PEO was completely removed and the membrane architecture was preserved. Annealing (slow cooling) produced a more developed folded surface, higher crystallinity, and greater shrinkage, whereas quenching (rapid cooling) retained a less ordered structure, lower shrinkage, and generally higher tensile strength and relative elongation. The highest tensile strength (4.12 ± 0.25 MPa) was obtained for the quenched 97.5/2.5 wt.% membrane, and the highest relative elongation (259 ± 16%) for the quenched 90/10 wt.% membrane. Exposure to nitric acid, potassium hydroxide, dimethyl sulfoxide, motor oil, and distilled water at 100 °C for 48 h caused no statistically significant changes in tensile strength or relative elongation. These results demonstrate that electrosprayed PTFE membranes offer tunable morphology and mechanical properties together with high chemical resistance, making them promising for aggressive-media filtration and separation.

## 1. Introduction

Electrospray is a technique for producing semipermeable porous membranes that are used in microelectronics as conductive tracks and transistors [1]; in the production of monodisperse powders [2], polymer microcapsules with prolonged drug release [3,4], and protective coatings [5]; in biocompatible scaffolds for tissue engineering [6]; and in the fabrication of solar cells [7], lithium-ion battery anodes and cathodes, and hydrogen storage systems [8]. In addition, electrospray is used to deposit polytetrafluoroethylene (PTFE) microparticles onto membrane surfaces to create superhydrophobic and superoleophobic coatings with a hierarchical structure, enabling highly efficient oil–water separation and demulsification [9]. Furthermore, this method enables the fabrication of Al/PTFE/NC composite microspheres with a self-reactive, three-dimensional, thermally conductive network, which are used as high-energy additives in energetic materials to enhance combustion pressure and energy release [10].

The broad range of applications of electrospray can be attributed to the unique combination of properties of the resulting materials, including resistance to aggressive media, the ability to maintain performance over a wide temperature range, excellent electrical insulating properties, and tissue biocompatibility [11,12,13]. Although the thermo-expanded method is still considered one of the most effective approaches for producing PTFE membranes with the required performance parameters, the past decade has seen a steady increase in interest in electrospray technology, which offers opportunities for fabricating materials with tunable surface morphology [14,15,16].

The electrospray/electrospinning technique is of particular interest because it enables the production of PTFE membranes composed of densely packed micrometer-sized spheres [17,18,19]. PTFE is a semicrystalline fluoropolymer characterized by high chemical and thermal stability, low surface energy, and excellent resistance to aggressive chemical media, making it an attractive material for porous membranes used in demanding environments [20]. However, the absence of solvents and the extremely high viscosity of the PTFE melt are essential for the direct production of porous PTFE membranes by these methods [21]. To overcome these limitations, the following strategy, comprising several main stages, is used. In the first stage, a spinning solution is prepared using aqueous suspensions with an average particle size of approximately 300 nm and a PTFE content of approximately 50 wt.% as the PTFE source [22,23]. High-molecular-weight poly (ethylene oxide) (PEO; ≈10^6^ g/mol) is typically used as an additive to the PTFE suspension; it is a water-soluble polymer with good fiber-forming properties. In the second stage, the resulting spinning solution is used to form a composite PTFE/PEO membrane, which has poor water resistance and low mechanical strength [24]. In the third stage, the composite PTFE/PEO membrane was subjected to thermal treatment at approximately 330–350 °C, which induced sintering of the PTFE particles and resulted in an open, interconnected porous structure, while PEO was completely eliminated through thermal decomposition [25,26,27].

The key properties determining the potential application of electrospray PTFE membranes are tensile strength and relative elongation. Maintaining these characteristics during prolonged exposure to aggressive media and elevated temperatures is particularly important for applications in the chemical industry, hydrogen energy, nuclear industry, and other demanding environments.

Because PTFE is a semicrystalline polymer, the mechanical properties of PTFE membranes are largely determined by the degree of crystallinity, which in turn depends on the heat treatment conditions, in particular the cooling rate of the membranes during the sintering stage [28,29]. To date, the effect of cooling rate on the tensile strength and relative elongation of membranes obtained by electrospray technique from spray solutions with different ratios of the sphere-forming polymer to PTFE microparticles has not been systematically studied. The available data are limited to membranes with fibrous morphology or single spray solution compositions [30,31]. Thus, the aim of the present work was to investigate the effect of heat treatment and exposure to aggressive media at high temperatures on the tensile strength and relative elongation of PTFE membranes obtained by electrospray technique at different PTFE/PEO ratios in spray solutions.

## 2. Materials and Methods

### 2.1. Materials

PTFE suspension F-4D (Technical specifications 6-05-1246-81, Galopolymer, Moscow, Russia), polyethylene oxide (PEO; molecular weight 0.6 × 10^6^ g/mol, Acros Organics, Geel, Belgium), nitric acid (HNO_3_, 10.3 mol/L, GOST R 4461-77, PK Acid S, Yekaterinburg, Russia), potassium hydroxide (KOH, 11.6 mol/L, GOST R 3118-77, Soda Chlorat, Berezniki, Russia), dimethyl sulfoxide (DMSO, Technical specifications 2635-114-44493179-08, Ekos 1, Moscow, Russia), motor oil (10W40, Rolf Energy, Obninsk, Russia), distilled water (H_2_O, GOST R 58144-2018, Sigma Tech, Shchelkovo, Russia), isopropyl alcohol (Technical specifications 2632-181-44493179-2014, Ekos 1, Moscow, Russia).

### 2.2. Preparation of Spray Solutions

To prepare the spray solutions 2.50 ± 0.01 g of PEO powder was dissolved in 47.50 ± 0.01 g of distilled water at 40 ± 5 °C for 2 h with continuous stirring in a water bath. The resulting 5 wt.% aqueous PEO solution was filtered through a 125 µm mesh filter (Holex Green, Shanghai, China). Before mixing, the PTFE suspension was stirred for 4 h and filtered through a 125 µm mesh filter (Holex Green, Shanghai, China). The PTFE suspension and aqueous PEO solution were then mixed using a ROTAX 6.8 mixer (Velp Scientifica, Usmate Velate, MB, Italy) for 8 h at room temperature. Four types of spray solutions were prepared for this study. The compositions of the solutions are shown in Table 1.

### 2.3. Spray Solution Characterization

The viscosity of the spray solutions was measured with an SV-10 viscometer (A&D, Tokyo, Japan). Electrical conductivity was measured with an InoLab Cond 7319 conductivity meter (WTW, Weilheim in Oberbayern, Germany) equipped with a TetraCon 325 measuring cell (WTW, Weilheim in Oberbayern, Germany). Both measurements were performed at 24 °C.

### 2.4. Electrospray Membrane Fabrication

Composite PTFE/PEO membranes were prepared using a multichannel electrospray setup (NIKA-1, Tomsk Polytechnic University, Tomsk, Russia), as previously described [32]. A photograph and a schematic representation of the multichannel electrospray system are shown in Figures S1 and S2 in the Supplementary Materials. An upper injector located above the assembly manifold was used to fabricate the membranes. The process parameters were as follows: 22G needle, 15 cm injector-to-collector distance, 200 rpm collector rotation speed, 1.5 mL/h flow rate, 40 kV applied voltage, 35 µA current, and 50 mm/s injector traverse speed. An aluminum cylinder (40 mm in diameter and 100 mm in length) covered with 40 µm thick aluminum foil was used as the collector. The electrospray process was carried out for 6 h. The ambient temperature and relative humidity were 26 °C and 24%, respectively.

### 2.5. Heat Treatment

Before heat treatment, the aluminum foil coated with the composite PTFE/PEO membrane was removed from the assembly manifold of a multichannel electrospray setup. The aluminum foil was then carefully separated from the PTFE/PEO composite membrane. The PTFE/PEO composite membrane samples were then placed in an electric chamber furnace (SNOL 3.5, TermIKS NPF, Moscow, Russia), where the composite PTFE/PEO membranes were heat-treated. The membranes were subjected to two heat-treatment regimes: quenching and annealing. During quenching, the composite PTFE/PEO membranes were heated in the furnace at a rate of approximately 1.7 °C/min to 330 °C, held at 330 °C for 10 min, and then cooled to room temperature by rapid removal from the furnace. The average cooling rate of the membranes was approximately 65 °C/min. During annealing, the composite PTFE/PEO membranes were heated at a rate of approximately 1.7 °C/min to 330 °C, held at 330 °C for 10 min, and then cooled to room temperature inside the furnace, after which the membranes were removed from the furnace. The average cooling rate of the membranes was approximately 0.4 °C/min.

### 2.6. Membrane Characterization

Membrane morphology before and after thermal treatment was examined by scanning electron microscopy (SEM) using a VEGA 3 SBH microscope (Tescan, Brno, Czech Republic). Samples were coated with a thin gold layer for 100 s using a DSR 1 magnetron sputtering system (Nano Structured Coatings Co., Tehran, Iran) in an argon atmosphere (50%) at a chamber pressure of 80 mTorr. The mean diameters of the structural elements were determined from five independent SEM images for each membrane type based on at least 250 measurements for each type of structural element, using ImageJ 1.38 software (National Institutes of Health, Bethesda, MD, USA).

Open porosity (*P*) was measured using the hydrostatic weighing method and calculated according to Equation (1) [33]. Isopropyl alcohol (Ekos-1, Moscow, Russia) was used as the liquid medium. Measurements were performed using an AF-224RCE analytical balance (Shinko Denshi, Tokyo, Japan) equipped with VIBRA AFDK hydrostatic weighing equipment (Shinko Denshi, Japan). Five independent measurements were performed for each membrane group.

(1) $$P=\frac{m_{1}-m}{m_{1}-m_{2}}\times 100\%$$

where *m* is the mass of the dry specimen weighed in air; *m*_1_ and *m*_2_ are the masses of the liquid-saturated specimen weighed in air and in the liquid, respectively.

The chemical structure of the PTFE membranes was studied by Fourier-transform infrared spectroscopy with attenuated total reflection mode (FTIR-ATR). The measurements were performed using a Tensor 27 IR spectrometer (Bruker, Kalkar, Germany) equipped with a PIKE MIRacle ATR accessory (PIKE Technologies, Madison, WI, USA) featuring a ZnSe crystal. The spectra were recorded in the range 370–4000 cm^−1^ with a resolution of 4 cm^−1^.

The degree of crystallinity of PTFE in the prepared membranes was calculated using Equation (2).

(2) $$X_{c}=\frac{H_{fm}}{H_{ft}}\times 100\%$$

where *H_fm_* is the experimental heat of fusion of PTFE, J/g; *H_ft_* is the heat of fusion of PTFE at 100% crystallinity, *H_ft_* = 60 ± 0.6 J/g [34].

Differential scanning calorimetry (DSC) was performed using a DSC Q2000 instrument (TA Instruments, New Castle, DE, USA) to determine the melting enthalpy of PTFE. The measurements were carried out under an argon atmosphere; the sample heating rate was 10 °C/min.

The relative shrinkage (*S_n_*) of the PTFE membranes obtained during thermal treatment of the composite PTFE/PEO membranes was calculated using Equation (3).

(3) $$S_{n}=\frac{S_{1}-S_{2}}{S_{1}}\times 100\%$$

where *S*_1_ is the area of the composite PTFE/PEO membranes before thermal treatment; *S*_2_ is the area of the PTFE membranes after thermal treatment.

### 2.7. Chemical Resistance Testing

The effect of the heat-treatment regime and exposure to aggressive media on the tensile strength and relative elongation of the PTFE membranes was evaluated using an Instron 3369 testing machine (Instron, Norwood, MA, USA) equipped with an Instron 2519-102 load cell (Instron, Norwood, MA, USA) at a crosshead speed of 10 mm/min. The blade-shaped specimens with a 10 mm long test part were obtained using a ZCP-020 cutting press (ZwickRoell GmbH & Co. KG, Ulm, Germany) equipped with a Type 4 cutting knife (ZwickRoell GmbH & Co. KG, Ulm, Germany) in accordance with ISO 37:2017 [35]. The specimens were cut perpendicular to the direction of collector rotation. Five specimens were tested for each membrane group. To study the effect of aggressive media on the tensile strength and relative elongation of the PTFE membranes, the following media were used: nitric acid; potassium hydroxide; dimethyl sulfoxide; engine oil; distilled water. The quenched PTFE membranes were immersed in each medium at 100 °C for 48 h.

### 2.8. Statistical Analysis

The data were analyzed using GraphPad Prism 9 software (GraphPad Software, Boston, MA, USA). Overall differences among the experimental groups were assessed using the non-parametric Kruskal–Wallis test. Pairwise comparisons between the quenched and annealed groups at each corresponding composition were performed using the Mann–Whitney U test. To account for multiple comparisons, *p*-values were adjusted using the Holm correction. Differences were considered statistically significant at *p* < 0.05.

## 3. Results and Discussion

**Figure 1:**
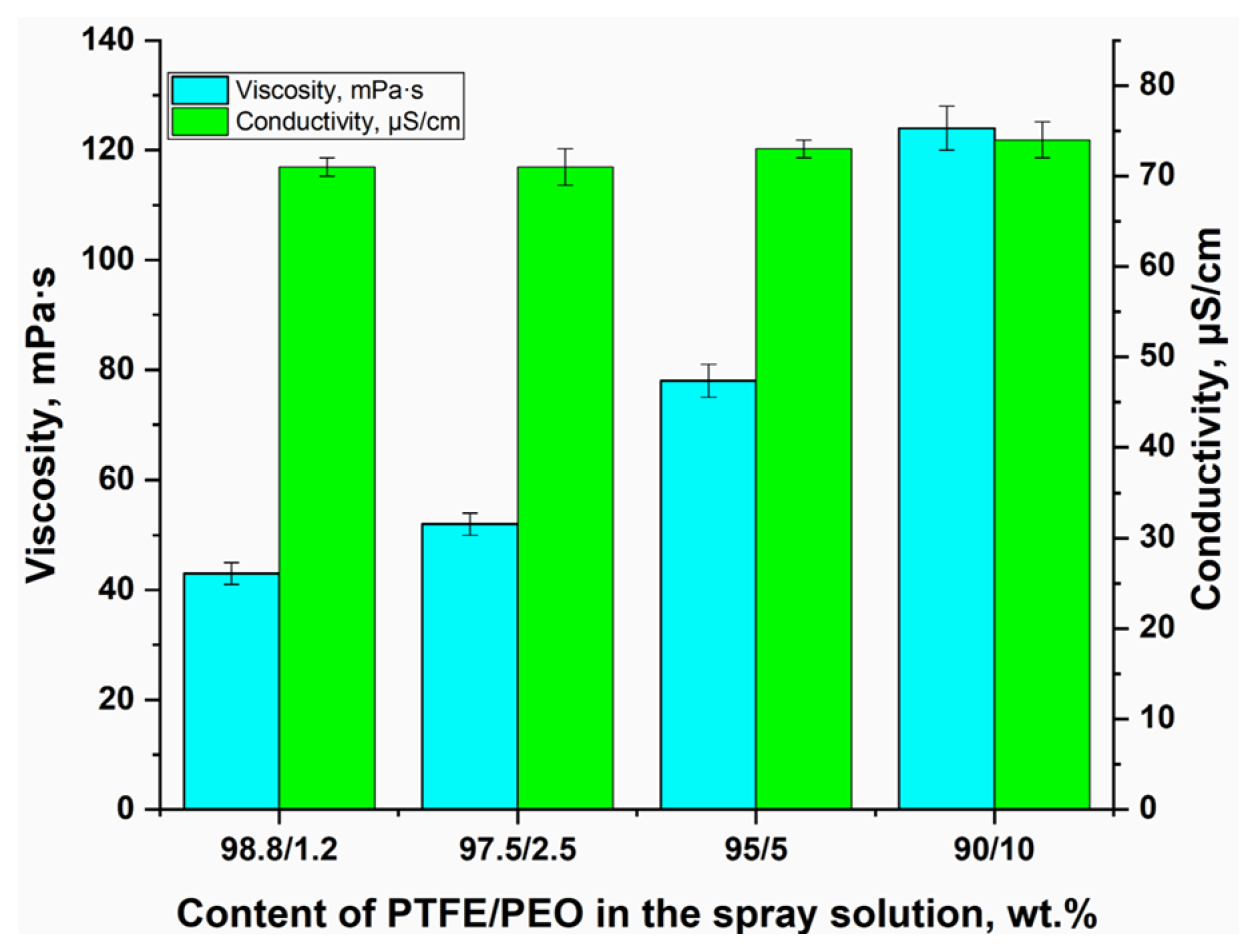
Viscosity and electrical conductivity of the spray solutions as functions of the composition of the spray solutions.

Figure 1 shows the effect of PTFE/PEO composition in the spray solution on viscosity and electrical conductivity.

The viscosity of the PTFE/PEO spray solutions increased with increasing PEO content, which is attributed to intermolecular interactions of the PEO macromolecules, resulting in the formation of temporary physical networks due to the entanglement of long polymer chains. As the PEO concentration increased, the number of such contacts increased, leading to increased resistance to flow and, consequently, to higher viscosity [36,37]. The absence of substantial changes in the electrical conductivity of the spray solution is due to the presence in the aqueous PTFE suspension of significant amounts (>5 wt.%) of nonionic surfactants used to stabilize the suspension. The high surfactant content, exceeding the critical micelle concentration, leads to the formation of electrically neutral micelles, which helps maintain the electrical conductivity of the spray solution [38].

**Figure 2:**
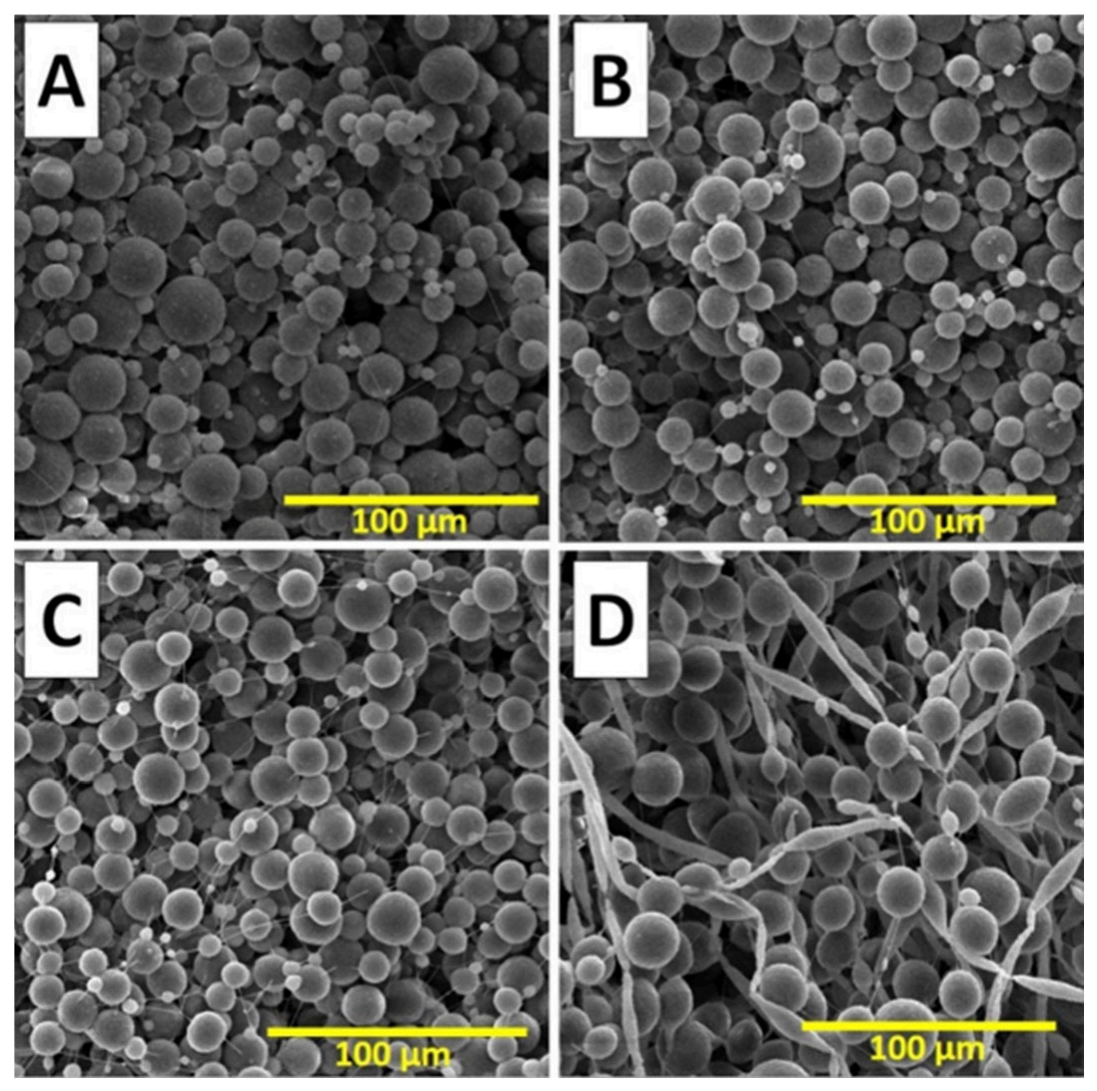
SEM images of the PTFE/PEO composite membranes before heat treatment, obtained from spray solutions with different compositions: (**A**) PTFE/PEO 98.8/1.2 wt.%; (**B**) PTFE/PEO 97.5/2.5 wt.%; (**C**) PTFE/PEO 95/5 wt.%; (**D**) PTFE/PEO 90/10 wt.%.

Figure 2 shows SEM images of membrane structures before heat treatment, obtained by the electrospray method from spray solutions with different compositions.

In the viscosity range of 43–78 mPa·s (PTFE suspension content: 95.0–98.8 wt.%), a structure consisting of spherical particles with an average diameter ranging from 3.2 to 3.7 µm is formed (Figure 2A–C). The formation of this structure can be attributed to the low viscosity, which does not favor the formation of a continuous jet but instead promotes its capillary breakup into multiple droplets [39]. The electrospray process is governed by a balance between the Coulomb forces that stretch the charged jet and the viscous forces that oppose its breakup. At low solution viscosity, the electrostatic repulsion of surface charges dominates, and the jet fragments into individual droplets to minimize surface energy [40]. When the viscosity reaches 124 mPa·s (PTFE suspension content: 90.0 wt.%), the system attains the threshold for fiber formation, and a transitional structure combining fibers (1.1 ± 0.2 µm) and spherical particles (3.6 ± 0.6 µm) is observed. In this regime, the increasing viscous forces begin to prevail over the capillary forces, ensuring jet stability and elongation into fibers. Thus, the critical viscosity window for forming a sphere-based structure is 43–78 mPa·s, and the transition to a fibrous structure occurs at viscosities above 124 mPa·s.

**Figure 3:**
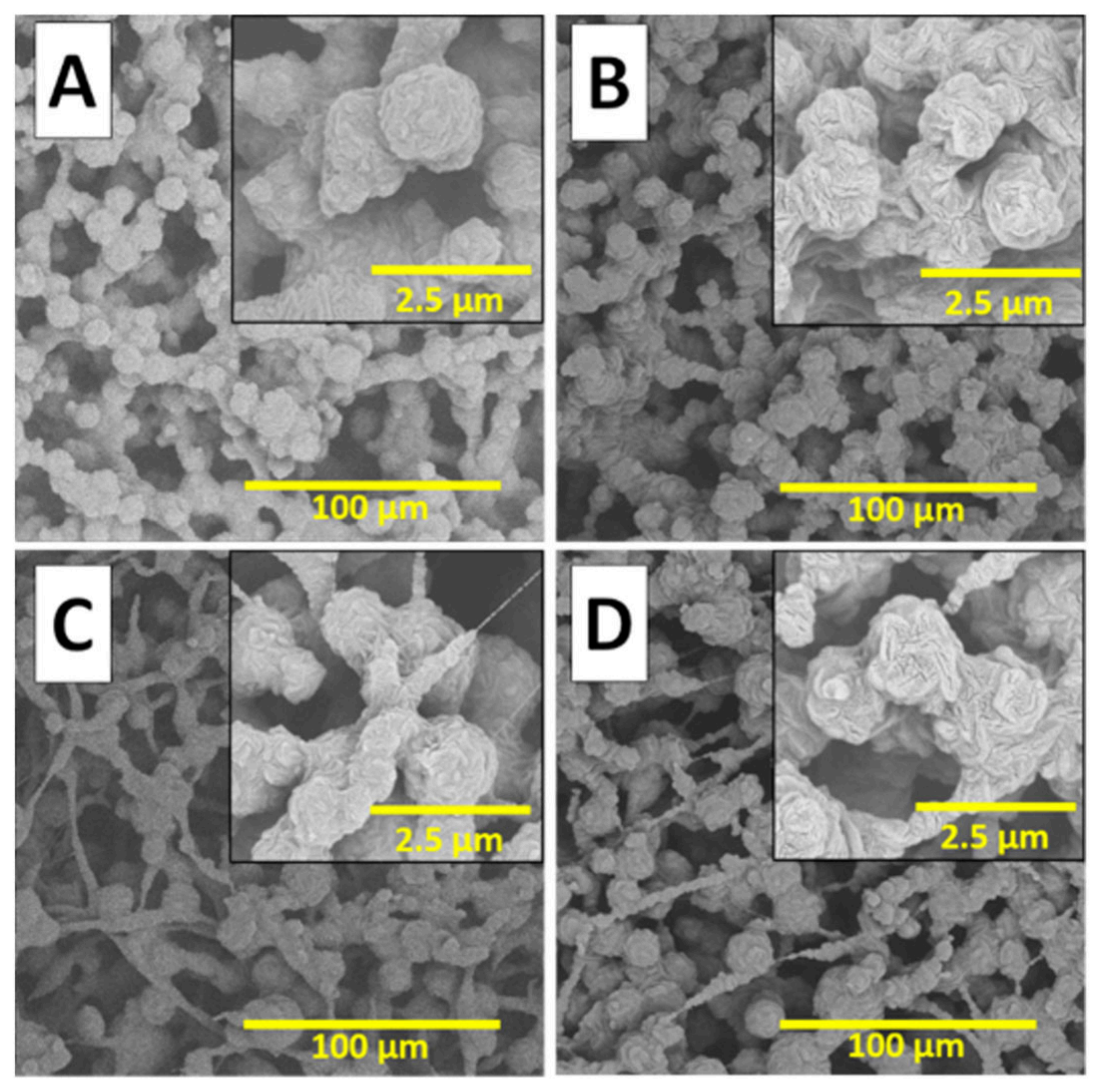
SEM images of PTFE membranes obtained by the electrospray method from spray solutions of different compositions, illustrating the effect of the heat treatment regime: (**A**) PTFE/PEO 97.5/2.5 wt.% (quenching); (**B**) PTFE/PEO 97.5/2.5 wt.% (annealing); (**C**) PTFE/PEO 90/10 wt.% (quenching); (**D**) PTFE/PEO 90/10 wt.% (annealing).

Figure 3 shows representative SEM images of PTFE membranes obtained by the electrospray method from spray solutions with different compositions, illustrating the effect of the heat treatment regime.

After heat treatment, the overall architecture of the membranes is preserved; however, changes in the morphology of the structural elements are observed depending on the heat treatment regime. The annealed samples are characterized by a well-developed wrinkled and folded surface microrelief with visible microcracks on the spherical polymer particles (Figure 3B,D). In contrast, the quenched membrane samples, subjected to rapid cooling, exhibit a smoother surface of the particles that form the membrane (Figure 3A,C).

**Figure 4:**
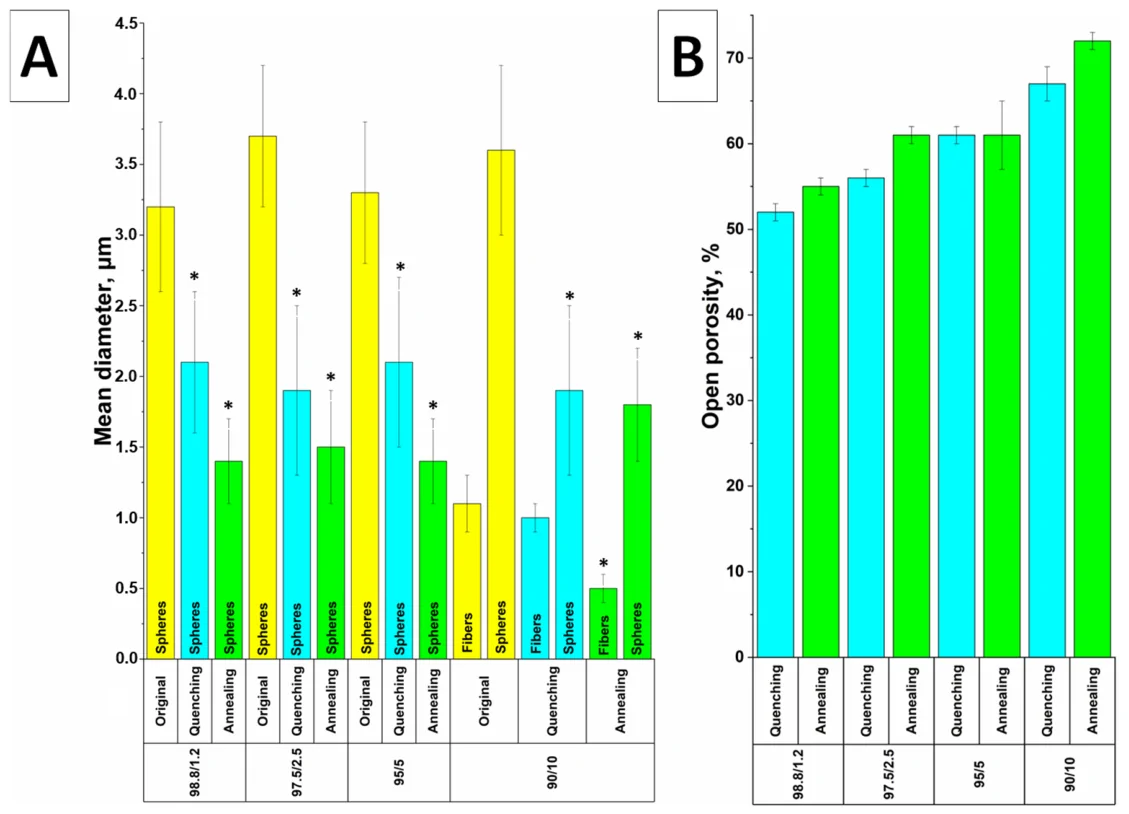
Mean diameter (spheres/fibers) (**A**) and open porosity (**B**) as functions of PTFE/PEO composition in the spray solution and heat treatment regime. *—*p* < 0.05 indicates a statistically significant difference compared with membranes without heat treatment.

Figure 4A shows that heat treatment decreased the average diameters of the spherical particles and, for the 90/10 wt.% sample, those of the fibers. For the 98.8/1.2, 97.5/2.5, and 95/5 wt.% PTFE/PEO samples, the initial average sphere diameters were 3.2 ± 0.6, 3.7 ± 0.5, and 3.3 ± 0.5 µm, respectively. Quenching reduced these diameters by 34%, 49%, and 36% (to 2.1 ± 0.5, 1.9 ± 0.6, and 2.1 ± 0.6 µm), whereas annealing reduced them to a greater extent by 56%, 59%, and 58% (to 1.4 ± 0.3, 1.5 ± 0.5, and 1.4 ± 0.3 µm). For the transitional 90/10 wt.% structure, the initial fiber and sphere diameters were 1.1 ± 0.2 and 3.6 ± 0.6 µm, respectively. Quenching decreased the fiber diameter by 9% to 1.0 ± 0.1 µm and the sphere diameter by 47% to 1.9 ± 0.6 µm; annealing decreased the fiber diameter by 55% to 0.5 ± 0.1 µm and the sphere diameter by 50% to 1.8 ± 0.4 µm. Overall, annealing produced greater diameter reductions than quenching, particularly for the fibers.

The open porosity in Figure 4B reflected the observed morphological differences. Annealed membranes generally exhibited higher open porosity than quenched membranes, except for the 95/5 wt.% composition, where the values were comparable. After annealing, the open porosity was 55 ± 1%, 61 ± 1%, 61 ± 4%, and 72 ± 1% for the 98.8/1.2, 97.5/2.5, 95/5, and 90/10 wt.% compositions, respectively; after quenching, it was 52 ± 1%, 56 ± 1%, 61 ± 1%, and 67 ± 2%.

The observed differences in the morphology of the structural elements of the membranes can be explained by differences in crystallization kinetics that depend on the cooling rate. During annealing (slow cooling), thermodynamically more favorable conditions are created for the rearrangement of PTFE macromolecules and their ordered packing, leading to the formation of a well-developed surface relief. During quenching (rapid cooling), the degree of structural rearrangement is limited because of the high heat removal rate; as a result, the macromolecules are fixed in a less equilibrated state, preventing the development of a pronounced folded surface morphology [41,42]. The reduction in the mean diameter of the spheres and fibers after heat treatment can be attributed to two main factors. First, during high-temperature sintering (at 330 °C), the PEO, which served as a binder component in the original PTFE/PEO composite membranes, is removed [43]. Second, the sintering process is accompanied by coalescence of PTFE particles, leading to their rearrangement and the formation of a denser structure with a smaller free volume [44,45].

**Figure 5:**
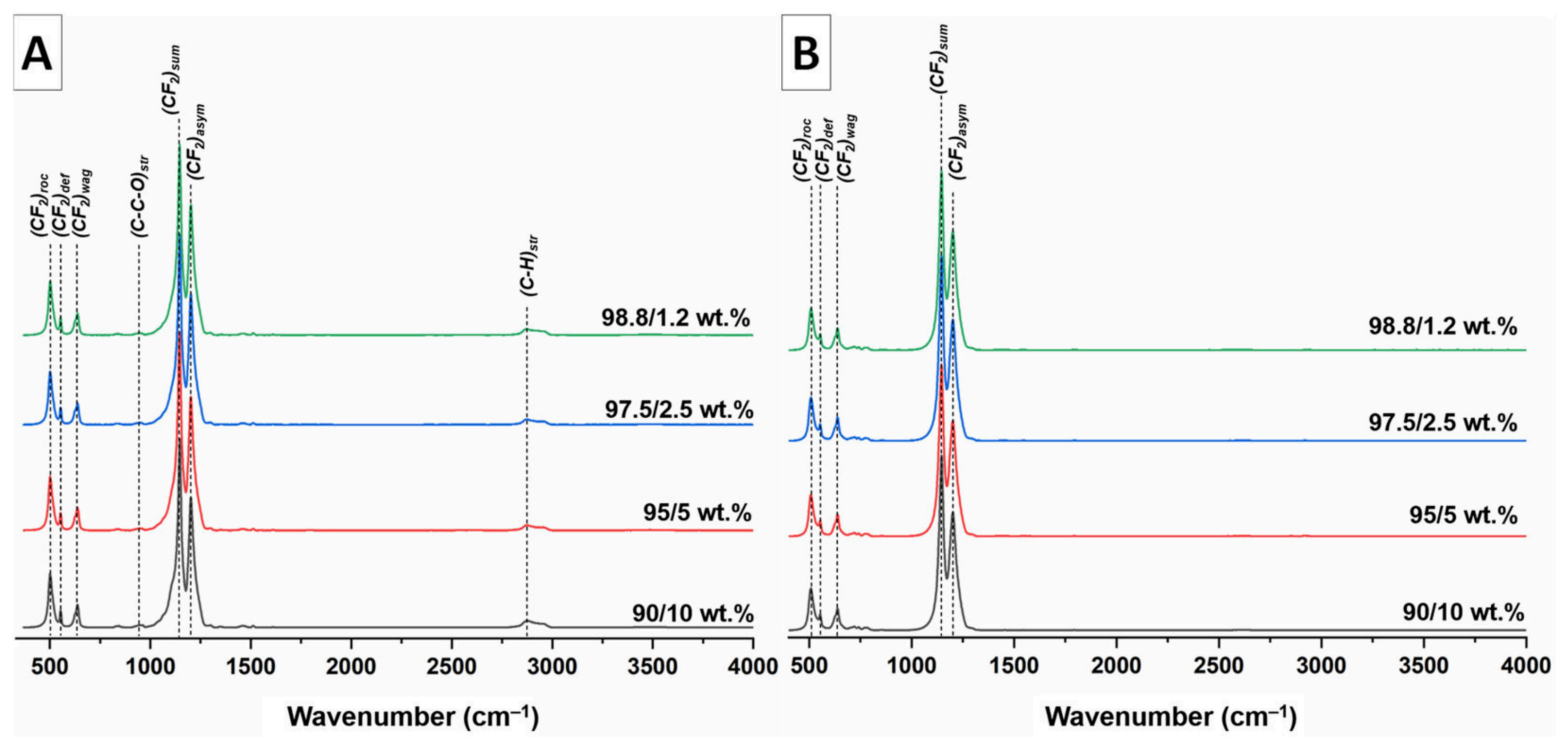
FTIR spectra of composite PTFE/PEO membranes before thermal treatment (**A**) and PTFE membranes after thermal treatment (**B**) as a function of the PTFE/PEO ratio in the spray solution.

Figure 5 shows the FTIR spectra of composite PTFE/PEO membranes before thermal treatment (Figure 5A) and PTFE membranes after thermal treatment (Figure 5B) as a function of the PTFE/PEO ratio in the spray solution. The FTIR spectra of composite PTFE/PEO membranes before thermal treatment show intense PTFE bands at 1147 and 1203 cm^−1^, corresponding to the symmetric and asymmetric stretching vibrations of the CF_2_ group, and at 512, 554, and 639 cm^−1^, corresponding to the vibrational and deformation modes of the CF_2_ group in PTFE [46].

Weak but distinguishable bands at 962 and 2880 cm^−1^ correspond to the rocking and symmetric stretching vibrations, respectively, of the CH_2_ group in PEO [47]. After thermal treatment, only PTFE bands remain in the FTIR spectra; no PEO bands are observed. Thus, after thermal treatment, PTFE membranes free of residual PEO are formed regardless of the PTFE suspension content in the spray solution. Previously, the removal of PEO from electrospun composite nonwoven membranes during sintering at ~380 °C was demonstrated by Zhao et al. [48].

**Figure 6:**
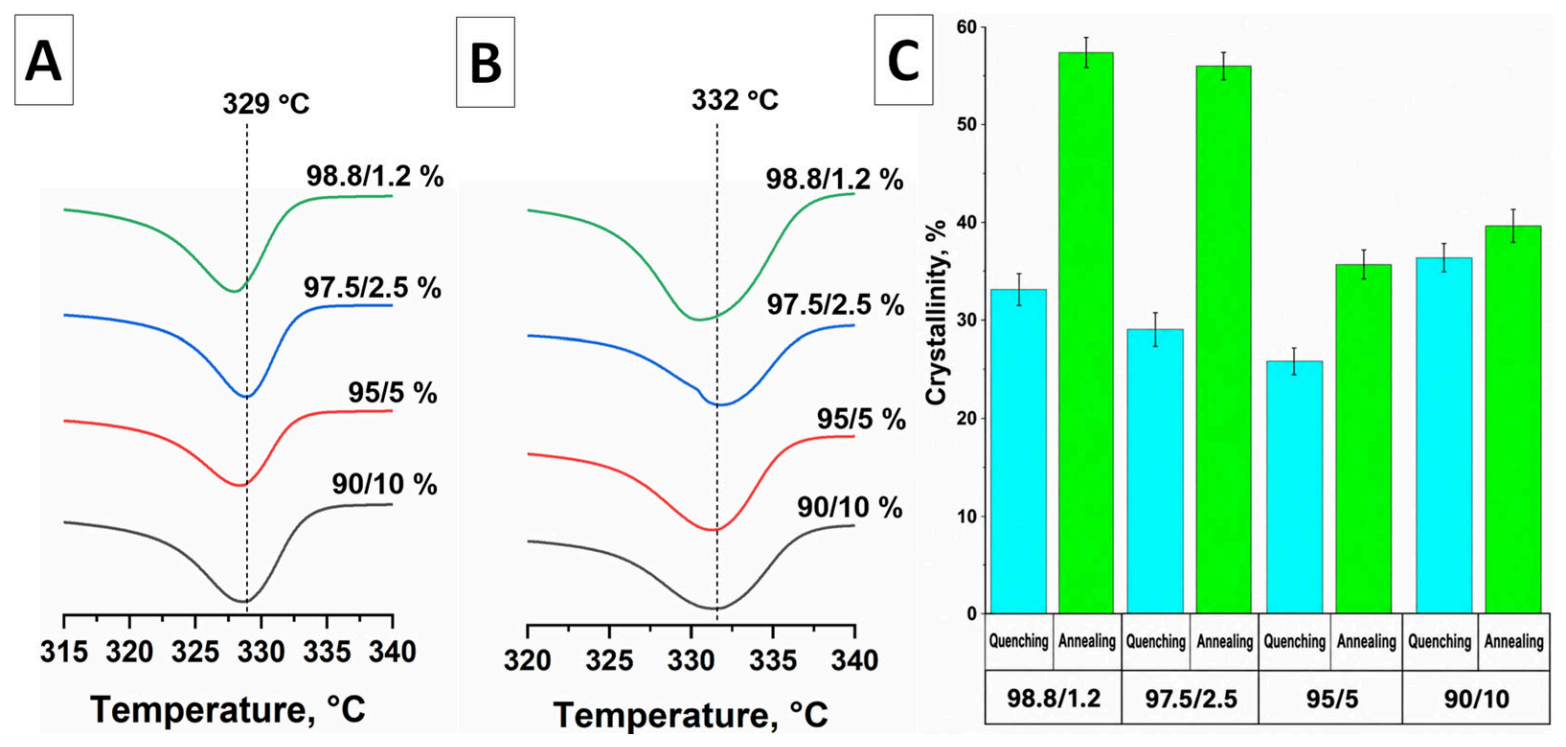
DSC thermograms of quenched (**A**) and annealed (**B**) PTFE membranes, and the degree of crystallinity of PTFE in the resulting membranes (**C**).

Figure 6A,B show DSC thermograms of the PTFE membranes as a function of PTFE/PEO ratio in the spray solution and heat treatment regime, while Figure 6C presents the degree of crystallinity of PTFE in the resulting membranes.

The DSC thermograms of quenched samples (Figure 6A) exhibit an intense melting peak at 329 °C, whereas for annealed samples (Figure 6B) the melting peak shifts to a higher temperature of 332 °C. The DSC results show that annealing provides a higher degree of crystallinity compared to quenching, regardless of the spray solution composition, which is attributed to the more ordered crystalline structure formed upon slow cooling (Figure 6C). The shift in the melting temperature to higher values (by 3 °C) for the annealed samples may be associated with an increase in crystallite size and a decrease in the number of crystal lattice defects, which is characteristic of annealing processes in semicrystalline polymers [49]. In the case of quenching, rapid cooling fixes a less ordered structure with a larger number of defects and smaller crystallites, leading to a decrease in both the melting temperature and the degree of crystallinity [50]. Thus, the heat treatment regime affects both the degree of crystallinity and the melting temperature of the PTFE membranes, with annealing promoting the formation of PTFE membranes with a higher degree of crystallinity.

**Figure 7:**
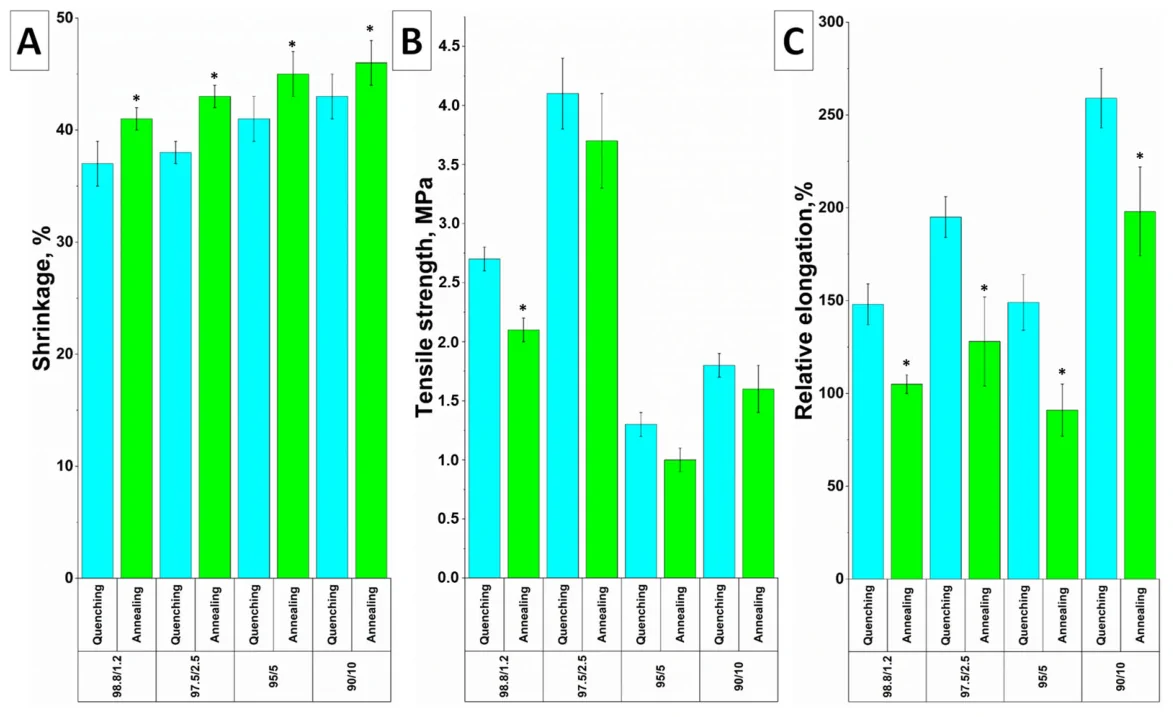
Shrinkage (**A**), tensile strength (**B**), and relative elongation (**C**) of PTFE membranes as a function of the PTFE/PEO ratio in the spray solution and the heat treatment regime. *—*p* < 0.05, statistically significant compared to the quenched membranes.

Figure 7 presents the results of the study on the effect of PTFE/PEO ratio in the spray solution and heat treatment regime on the shrinkage, tensile strength, and relative elongation of the PTFE membranes.

The results presented in Figure 7A show that all investigated PTFE membrane samples exhibit substantial shrinkage, with values ranging from 37 ± 2% to 46 ± 2% depending on the spray solution composition and heat treatment regime. A clear trend towards lower shrinkage is observed with increasing PTFE content in the spray solution (i.e., decreasing PEO fraction): for the 90/10 wt.% composition, shrinkage is 43 ± 2% (quenching) and 46 ± 2% (annealing), whereas for the 98.8/1.2 wt.% membranes it decreases to 37 ± 2% (quenching) and 41 ± 1% (annealing). Comparison of the two heat treatment regimes showed that quenched membranes consistently exhibited lower shrinkage than annealed samples across all studied compositions. This difference can be attributed to the different cooling rates and their influence on the resulting crystalline structure. During annealing, slow cooling promotes crystallite growth, increases the degree of crystallinity, and reduces crystal lattice defects [49]. This leads to the formation of a densely packed structure with smaller free volume and, consequently, greater shrinkage. In contrast, quenching fixes a less ordered structure with lower crystallinity and smaller crystallite size, preserving a larger free volume [50]. Thus, membrane shrinkage decreases with increasing PTFE fraction in the spray solution, and quenching enables lower shrinkage compared to annealing due to the retention of a less perfect crystalline structure, which may be associated with a less compact structure.

The mechanical properties of the membranes (Figure 7B,C) demonstrated a pronounced dependence on both the spray solution composition and the heat-treatment regime. The highest tensile strength was observed for membranes with a PTFE/PEO ratio of 97.5/2.5 wt.%: 4.12 ± 0.25 MPa after quenching and 3.80 ± 0.44 MPa after annealing, which were higher than those obtained for the other investigated compositions. The lowest tensile strength values were observed for membranes with a PTFE/PEO ratio of 95/5 wt.%: 1.20 ± 0.05 MPa after quenching and 1.00 ± 0.10 MPa after annealing. For all investigated compositions, the tensile strength was higher after quenching than after annealing. The relative elongation was highest for the samples with the highest PEO content (90/10 wt.%) after quenching (259 ± 16%) and lowest for the 95/5 wt.% membranes after annealing (91 ± 14%). Overall, the higher tensile strength and relative elongation observed after quenching compared with annealing may be attributed to the higher cooling rate, which limits crystallization and results in a lower degree of crystallinity [31,51].

**Figure 8:**
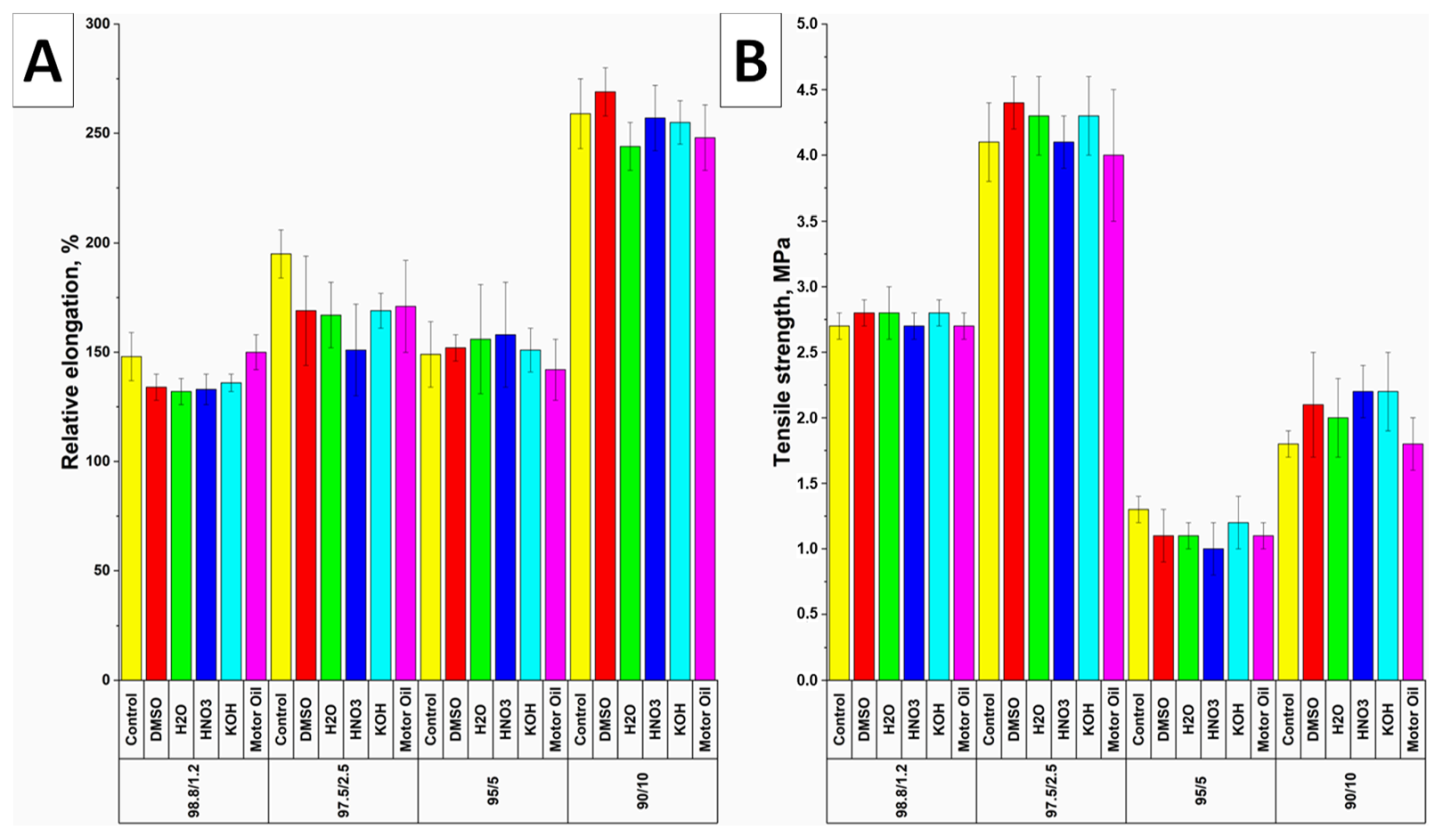
Relative elongation (**A**) and tensile strength (**B**) of quenched PTFE membranes before and after exposure to aggressive media as a function of the PTFE/PEO ratio in the spray solution. *p* < 0.05 indicates a statistically significant difference compared with control membranes not exposed to aggressive media.

Among the obtained samples, quenched PTFE membranes were selected for further studies because they generally exhibited higher tensile strength and elongation than the annealed membranes. The results of the study on the influence of aggressive media on the tensile strength and relative elongation of quenched PTFE membranes as a function of spray solution composition are presented in Figure 8.

The results indicate that no statistically significant differences in tensile strength or relative elongation were observed between the PTFE membranes before and after exposure, regardless of the PTFE suspension content in the spray solution or the type of aggressive medium (Figure 8A,B). The sample dimensions also remained unchanged. The preservation of the mechanical properties of the PTFE membranes can be attributed to the full thermal degradation of PEO (Figure 5B) [43]. Consequently, subsequent exposure to aggressive media occurs primarily on PTFE, which exhibits high chemical inertness [48,52]. The high chemical inertness of PTFE is attributed to the strong C-F bonds and the dense helical conformation of the macromolecules, which effectively shield the carbon backbone [30], ensuring the retention of the original mechanical properties of the PTFE membranes.

The results indicate that electrospray PTFE membranes exhibit high chemical resistance to various aggressive agents. This opens up the possibility of using such membranes in a range of applications, primarily for the filtration of aggressive media and the separation of emulsions in the chemical, food, and pharmaceutical industries, as well as in microelectronics production and in fuel cells, electrolyzers, and batteries [26,53]. These applications will be the subject of further research.

## 4. Conclusions

In this work, the effects of the mass ratio of the polytetrafluoroethylene suspension to the polyethylene oxide solution in the spray solution, the heat treatment regime, and exposure to aggressive media at elevated temperatures on the shrinkage, tensile strength, and relative elongation of PTFE membranes obtained by electrospray technique were investigated. Decreasing the PTFE suspension content in the spray solution and correspondingly increasing the PEO content increased the solution viscosity from 43–78 to 124 mPa·s, leading to a transition from electrospraying to electrospinning and the formation of a structure composed of fibers and spheres. Quenching resulted in lower shrinkage than annealing, whereas the tensile strength and elongation were generally higher after quenching. After thermal treatment, shrinkage ranged from 37 ± 2% to 46 ± 2%; quenching consistently produced lower shrinkage than annealing. The highest tensile strength was obtained for the 97.5/2.5 wt.% composition, reaching 4.1 ± 0.3 MPa after quenching, while the highest relative elongation was 259 ± 16% for the quenched 90/10 wt.% membrane. After exposure of the PTFE membranes to concentrated mineral acids and alkalis, motor oil, and distilled water at 100 °C for 48 h, no statistically significant differences were detected in their tensile strength or relative elongation, supporting their high chemical resistance under the tested conditions and their potential use for aggressive media filtration.

## Figures and Tables

**Table 1:** Composition of the spray solutions used for membrane fabrication by electrospraying and the PTFE/PEO content of the spray solutions relative to the dry matter.

Composition of the Spray Solutions, wt.%		PTFE/PEO Content in the Spray Solutions Relative to Dry Matter, g	
PTFE Suspension	5 wt.% Aqueous PEO Solution	PTFE	PEO
98.8 ± 0.1	1.2 ± 0.1	23.98 ± 0.01	0.03 ± 0.01
97.5 ± 0.1	2.5 ± 0.1	23.67 ± 0.01	0.05 ± 0.01
95.0 ± 0.1	5.0 ± 0.1	23.07 ± 0.01	0.10 ± 0.01
90.0 ± 0.1	10.0 ± 0.1	21.85 ± 0.01	0.22 ± 0.01

## Data Availability

The original data presented in this study are openly available on Google Drive at https://clck.ru/3WPNDv (accessed on 1 October 2026).

## Supplementary Materials

The following supporting information can be downloaded at: https://www.mdpi.com/article/10.3390/polym18202504/s1, Figure S1: Schematic of the multi-channel electrospray system NIKA-1 (TPU, Tomsk, Russia); Figure S2: Multi-channel electrospray setup NIKA-1 (TPU, Tomsk, Russia); Figure S3: SEM images of PTFE membranes obtained by electrospray technique as a function of the PTFE/PEO ratio in the spray solution after thermal treatment (quenching): (a) 98.8/1.2 wt.%; (b) 97.5/2.5 wt.%; (c) 95/5 wt.%; (d) 90/10 wt.%; Figure S4: SEM images of PTFE membranes obtained by electrospray technique as a function of the PTFE/PEO ratio in the spray solution after thermal treatment (annealing): (a) 98.8/1.2 wt.%; (b) 97.5/2.5 wt.%; (c) 95/5 wt.%; (d) 90/10 wt.%.

## Abbreviations

The following abbreviations are used in this manuscript:

PTFE	Polytetrafluoroethylene
PEO	Polyethylene oxide
