# Supplementary material for: Electrospray Polytetrafluoroethylene Membranes: Effect of Composition and Heat Treatment on Mechanical Properties and Chemical Resistance
Source: Polymers (Basel). 2026 Oct 13;18(20):2504. doi: 10.3390/polym18202504 (PMC13709095; doi:10.3390/polym18202504)
Supplement: Supplementary file 1 [file polymers-18-02504-s001.zip › polymers-4546968-supplementary.pdf]

Electrospray Polytetrafluoroethylene Membranes: Effect of Composition and Heat Treatment on Mechanical Properties and Chemical Resistance

Evgeniy Melnik [1], Vladimir Botvin [2], Kirill Shirin [2], Valery Svetlichnyi [3], Aleksandra Golubovskaya [3], Vyacheslav Buznik [2,4], Andrey Yaroslavtsev [4] and Evgeny Bolbasov [1,*]

[1] Additive Technologies Center, National Research Tomsk Polytechnic University, Tomsk 634050, Russia
[2] Faculty of Chemistry, National Research Tomsk State University, Tomsk 634050, Russia
[3] Laboratory of Advanced Materials and Technology, National Research Tomsk State University, Tomsk 634050, Russia
[4] Kurnakov Institute of General and Inorganic Chemistry of the Russian Academy of Sciences, Moscow 119991, Russia
* Correspondence: ftoroplast@tpu.ru; Tel.: +7-960-978-23-15

Supplementary figures:

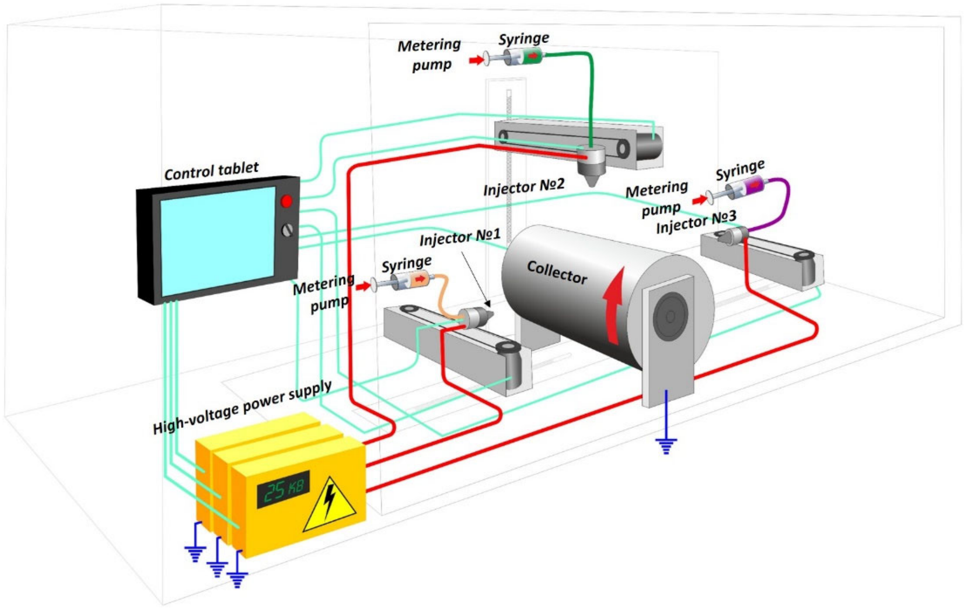

Figure S1. Schematic of the multi-channel electrospray system NIKA-1 (TPU, Tomsk, Russia)

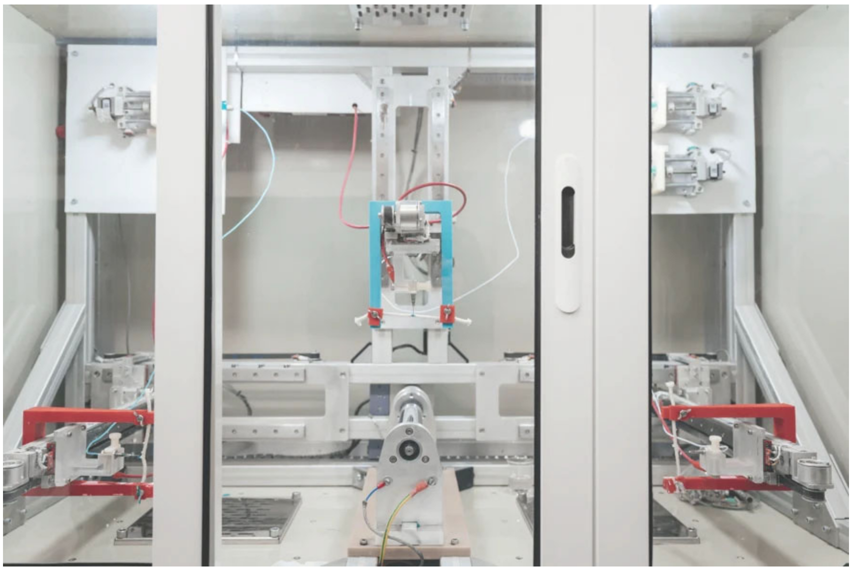

Figure S2. Multi-channel electrospray setup NIKA-1 (TPU, Tomsk, Russia)

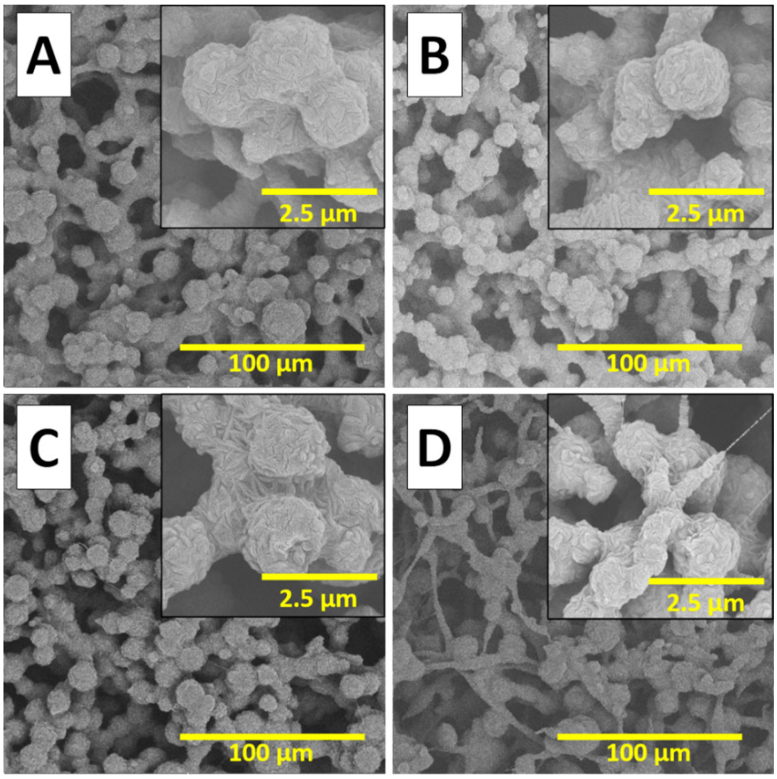

Figure S3. SEM images of PTFE membranes obtained by electrospray technique as a function of the PTFE/PEO ratio in the spray solution after thermal treatment (quenching): (a) 98.8/1.2 wt.%; (b) 97.5/2.5 wt.%; (c) 95/5 wt.%; (d) 90/10 wt.%.

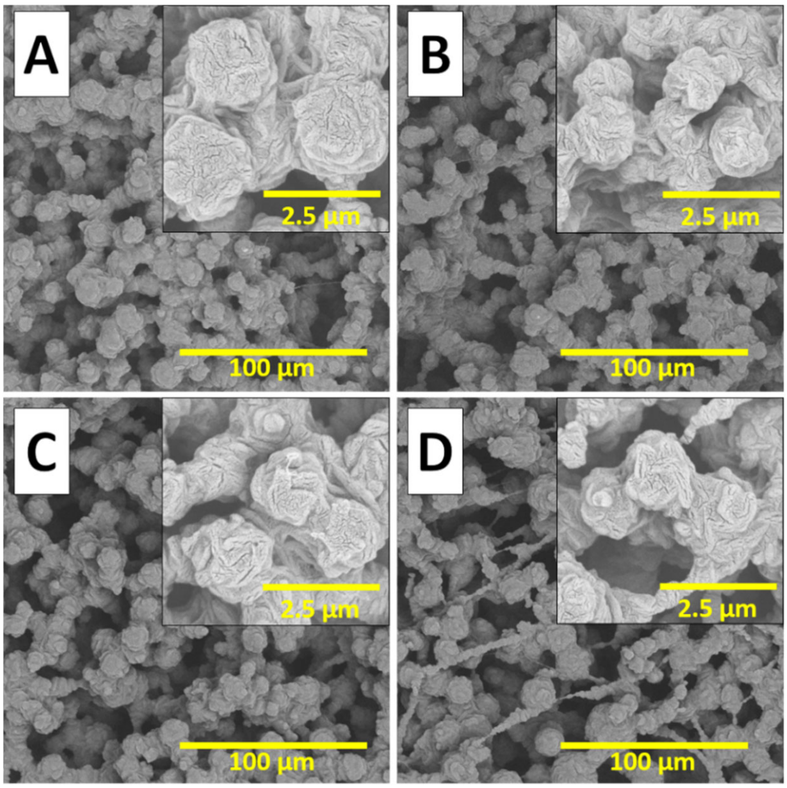

Figure S4. SEM images of PTFE membranes obtained by electrospray technique as a function of the PTFE/PEO ratio in the spray solution after thermal treatment (annealing): (a) 98.8/1.2 wt.%; (b) 97.5/2.5 wt.%; (c) 95/5 wt.%; (d) 90/10 wt.%.
